# Measurement, manipulation and modeling of brain-wide neural population dynamics

**DOI:** 10.1038/s41467-020-20371-1

**Published:** 2021-01-27

**Authors:** Krishna V. Shenoy, Jonathan C. Kao

**Affiliations:** 1 Department of Electrical Engineering, Stanford University, Stanford, CA 94305 USA; 2 Department of Bioengineering, Stanford University, Stanford, CA 94305 USA; 3 Department of Neurobiology, School of Medicine, Stanford University, Stanford, CA 94305 USA; 4 Wu Tsai Neuroscience Institutes, Stanford University, Stanford, CA 94305 USA; 5 Bio-X Institute, Stanford University, Stanford, CA 94305 USA; 6 Howard Hughes Medical Institute (HHMI) at Stanford University, Stanford, CA 94305 USA; 7 Department of Electrical and Computer Engineering, University of California, Los Angeles, Los Angeles, CA USA; 8 Neurosciences Program, University of California, Los Angeles, Los Angeles, CA USA

**Keywords:** Neuroscience, Computational neuroscience, Motor control

## Abstract

Neural recording technologies increasingly enable simultaneous measurement of neural activity from multiple brain areas. To gain insight into distributed neural computations, a commensurate advance in experimental and analytical methods is necessary. We discuss two opportunities towards this end: the manipulation and modeling of neural population dynamics.

Neural circuits comprise networks of individual neurons that perform sensory, cognitive, and motor functions. Neuronal biophysics, together with these circuits, give rise to neural population dynamics, which express how the activity of the neural population evolves through time in principled ways. Neural population dynamics provide a framework for understanding neural computation. Prior studies have modeled neural population dynamics to gain insight into computations involved in decision-making, timing, and motor control^1^. Here, we present emerging opportunities for new experiments and analyses that use a dynamical systems framework to better understand brain circuits, how they interact, and how they relate to behavior.

The simplest model of neural population dynamics is a linear dynamical system (LDS). An LDS (Fig. 1a) is described by a *dynamics* equation (**x**(t + 1) = **Ax**(t) + **Bu**(t)) and an *observation* equation (**y**(t) = **Cx**(t) + **d**). Typically, **y**(t) reflects experimental measurements, such as a vector where each element is the number of action potentials fired by a neuron in a brief time bin (e.g., 10 ms). The vector **x**(t) is a “neural population state” that captures information in **y**(t). This neural population state can be thought of as a representation of the dominant activity patterns in the experimental neural recordings. Typically, **x**(t) is an abstract representation in a low-dimensional subspace (or manifold) found via dimensionality reduction^2^ (Fig. 1b, neural state), reflecting that the neural activity is correlated and the dominant patterns can be described by a relatively small number of variables. The neural population state can also represent the activity of each neuron in the original dimensionality of the measured data (e.g., 100D if 100 neurons). The observation equation relates the observed action potentials (**y**(t)) to the neural population state (**x**(t)) through an observation matrix (**C**). The vector, **d**, is a constant offset (e.g., to model baseline firing). The neural population state moves through neural state space, constituting a neural population trajectory. The dynamics equation expresses how the neural population state (**x**(t)) progresses through time as a function of a dynamics matrix (**A**), an input matrix (**B**) and inputs (**u**(t)) from other brain areas and sensory pathways (Fig. 1c, neural dynamics). The neural population state and its dynamics are informative of behavior^3,4^.

**Fig. 1:**
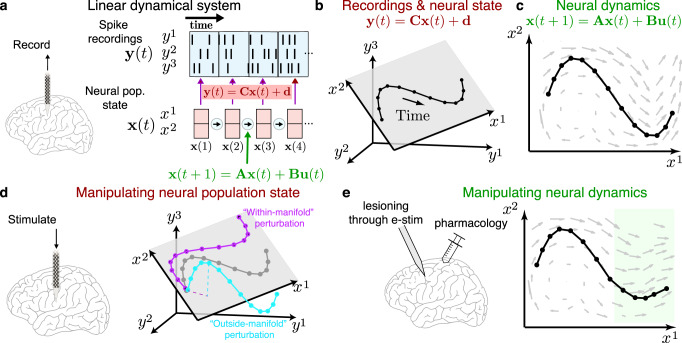
Overview of neural dynamics and manipulations. **a** Linear dynamical system using neural recordings. Binned spiking activity, **y**(t), relates to a latent neural population state, **x**(t), that evolves according to linear dynamics, **A**, with inputs from other cortical areas, **Bu**(t). The neural population state is typically a low-dimensional trajectory, and the dynamics can be conceptualized as a flow field. **b** In many cases, the neural state can be represented as a low-dimensional trajectory in a subspace of the higher-dimensional recordings. **c** In this subspace, the neural state evolves according to neural dynamics, which define a flow field. In a LDS, the dynamics of **A** can be contractive, expansive, rotational, or a fixed point. Inputs may cause the flow field to exhibit more complex motifs, such as shown in this panel. Generally, dynamics and dimensionality reduction can also be modeled to be nonlinear. **d** By electrical or optogenetic stimulation, or applying perturbations to the sensory-behavior or force-behavior relationship, it is possible to make perturbations to the neural state, **x**(t). These perturbations can be “within-manifold”, which perturbs the neural state along its natural modes, or they can be “outside-manifold,” which perturbs the neural state along dimensions outside the plane spanned by x^1^ and x^2^. **e** The neural dynamics (flow field, **A**) can also be perturbed, for example by applying pharmacology or lesioning the circuit. The neural state changes as a result of the changing dynamics (highlighted in green). Panels **a** and **b** are modified by J. C. Kao and appeared in Pandarinath and colleagues 2018^38^.

LDSs and nonlinear dynamical systems models^5,6^, paired with measurements from populations of individual neurons from one or more brain areas^7–14^, have produced new insights into the putative computational functions being performed^1^. Here we discuss emerging opportunities to expand dynamical systems insights into brain function. We focus on (1) manipulating neural dynamics and states, enabling future experiments to causally probe neural dynamics and their roles in computation, and (2) modeling multi-area dynamics spanning multiple brain areas, which leverages brain-wide measurements enabled by new large-scale electrophysiological neural recording methods^9,10^.

## Manipulating neural dynamics

To date, dynamical systems studies have primarily modeled neural population recordings during behaviors. By building on work to perturb the neural circuit, future experiments may further elucidate properties of the circuit and determine causal circuit roles. Two important opportunities are to (1) casually perturb the neural activity, which is present in the neural circuitry, and (2) causally alter the neural circuit dynamics, which reflect the neural circuitry.

First, causally perturbing **x**(t) and observing how the neural circuit dynamics counteract this perturbation helps us learn more about **A**^1^. We can also gain insight by perturbing inputs from other brain areas, **u**(t)^15^. There are several ways to casually perturb neural activity, including electrical microstimulation^16^ and optogenetic stimulation^17,18^. Neural activity can also be causally perturbed through task manipulations^4^, including changing visual targets during a computation^19^ and/or during behavior^20^, and changing the sensory-behavior relationship^21,22^. An emerging challenge is to perturb a population of neurons with spatio-temporal patterns that activate the circuit ‘within-manifold.’ These within-manifold perturbations (Fig. 1d) alter neural activity in a manner consistent with the circuit’s natural activations^17,18,23^. Within-manifold perturbations can therefore be viewed as displacements of the neural state in the activity’s low-dimensional manifold (Fig. 1d). In contrast, an ‘outside-manifold’ perturbation^24^ would result in neural activity that the circuit would not naturally exhibit. In general, perturbations such as optogenetic or electrical stimulation that do not explicitly consider the low-dimensional manifold of the circuit are outside-manifold. Outside-manifold perturbations may be informative, for example, by revealing interesting dynamics in unexplored dimensions previously unaccounted for. We highlight that precise within-manifold perturbation at millisecond precision will likely lead to significant insights on computation through dynamics, enabling experimenters to causally test the impact of the neural state on behavior^4^. However, these types of perturbations are challenging because they generally require the ability to deliver precise excitation and inhibition to individual neurons at millisecond precision to induce a desired change in neural state. Overall, causal perturbation of **x**(t) and **u**(t), and examination of their effects on behavior, may identify dimensions that are causally linked to behavior and learning, and how neural dynamics respond to both natural and unusual perturbations in the circuit.

A related second emerging opportunity is to alter the dynamics matrix **A** by experimentally changing the neural circuit. This can be achieved by local infusion (e.g., muscimol, chemogenetics) or systemic delivery (e.g., oral methylphenidate) of transiently acting pharmacological agents, altering local activity by delivering energy (e.g., continuous optogenetic stimulation, cooling^25^, transcranial stimulation, focal ultrasound stimulation), or lesioning (which can be performed in a variety of ways). Modifying **A** may have diverse effects. For example, cooling appears to slow down trajectories within-manifold^4^, while lesioning may change the manifold and its dynamics by permanently removing neurons from a circuit. Pharmacological agents may have brain-wide changes in dynamics across multiple areas, or more local changes if a local acting agent is used (e.g., muscimol). Understanding how modifications to the neural circuit’s dynamics influence behavior will be important for future treatments of, and recovery from, neurological and psychiatric disorders.

## Models of large scale, brain-wide neural population dynamics

**Fig. 2:**
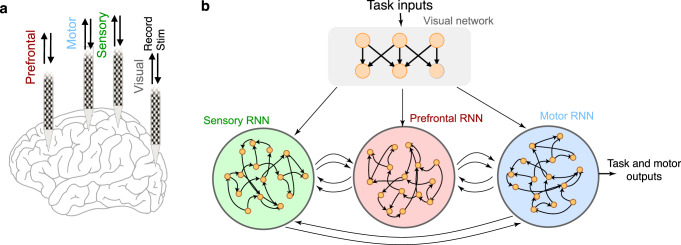
Using neural networks to model multi-area computation. **a** Multi-area, brain-wide electrical recording and stimulation of neural activity is rapidly becoming possible, and these data require new analyses and modeling to provide new scientific insights and theories of neural computation. High-density NeuroPixel electrodes are shown inserted in four locations, and many additional insertions are possible in both cortical and subcortical regions^9^. Each insertion can access several different brain areas (M). Thus the size of the data is proportional to N × M. The proportionality constant depends on the type of neural recordings, with full broadband data including low-frequency local field potentials (LFPs) and action potentials (APs, or spikes) requiring the fastest sampling. For stimulation, it is possible to stimulate arbitrary waveforms on each electrode in each area and thus there are enormous combinatorial possibilities^39^. Single and two photon optical imaging of genetically encoded calcium indicators and voltage indicators, and optogenetic neural modulation, are also widely used but are not shown for simplicity. **b** Multi-area RNNs can be trained to model each of these insertions. Visual areas may be modeled via convolutional neural networks^40, 41^ or related artificial networks that incorporate recurrence. Areas like the prefrontal and motor cortex are typically modeled by RNNs^6,30^. Regularizations may be employed so that activity within an RNN area resembles those recorded from each electrode array recordings.

Advanced neural technologies enable recording from many thousands of neurons across multiple interacting brain areas^10^ (Fig. 2a). There are several modeling challenges and opportunities for dynamical systems analyses, including how to increase the modeling capacity of dynamical systems models, denoising neural data from multiple areas^16^, incorporating physiological constraints into dynamical systems models, and interpreting dynamical models to generate new hypotheses for neural computation. We focus on one particular opportunity where we believe dynamical systems modeling is important: modeling distributed brain-wide computations that span multiple areas, each playing a distinct and critical role.

In cognitive and motor tasks, task-related activity arises in multiple brain areas. Evidence shows many brain areas are necessary to perform tasks at high performance, indicative that distributed computation between areas plays a critical role^26^. Multi-area recordings and analyses ought to enable new insights into how distributed computation occurs across brain areas, addressing key questions including the following. What are the neural dynamics within each area, and how do these relate to the overall distributed computation and behavior? How are neural representations similar and different across brain areas, and what are the computational benefits of these representations? What types of information are conveyed between brain areas through inter-area connections? How are dynamics and inter-area connections coordinated? There are significant computational challenges to answering these questions. Even with multi-area recordings, information about axonal connectivity between areas may generally be unknown, requiring new computational approaches to model multi-area interactions.

Traditionally, inter-area communication between cortical areas is thought to rely on temporal coordination and communication through coherences^27^. With new multi-area datasets, recent studies viewing cortical computations as low-dimensional systems have resulted in new hypotheses for inter-area communication and multi-area dynamics. To help conceptualize multi-area dynamics, consider a didactic and oversimplified example of coupled LDSs for two areas. Here, one LDS models area 1 (subscript 1) and another LDS models area 2 (subscript 2). They are coupled through axonal projections: **x**_1_(t + 1) = **A**_**1**_**x**_1_(t) + **B**_1_**u**_1_(t) + **B**_2-to-1_**x**_2_(t) and **x**_2_(t + 1) = **A**_**2**_**x**_2_(t) + **B**_2_**u**_2_(t) + **B**_1-to-2_**x**_1_(t). **B**_1-to-2_ maps the neural state from area 1 as inputs to area 2, and vice versa for **B**_2-to-1_. Although axonal projections may not be recorded between areas, dynamical systems models using recordings from both areas 1 and 2 can provide insight into the information communicated between areas. In particular, **B**_1-to-2_ can be thought of as a communication subspace (CS) that selectively extracts features of **x**_1_ to propagate to **x**_2_, summarizing the role of inter-area axonal projections^11,12,28^. The CS may not be aligned with neural dimensions of highest variance (such as the principal components) but may instead communicate activity along low variance dimensions that are necessary for downstream computation. By conceptualizing inter-area communication as this matrix multiplication, the CS builds on the principle of “output-null” spaces: information not necessary for downstream areas may be attenuated through alignment with the effective nullspace of the CS matrix. This phenomenon was initially observed for preparatory activity in PMd, which is attenuated in M1, likely due to its partial alignment with an output-null space^7^.

There are challenges towards using dynamical system models to study multi-area computation. One challenge is to design models that couple within-area dynamics with inter-area connections. For example, what dynamical computations are performed along dimensions that are either orthogonal or read out by a CS? An important area of future research will be developing systems identification techniques to learn parameters of coupled dynamical systems from multi-area neural recordings. Second, it could be that key inputs to a brain area are not recorded. Future techniques should consider how to address these missing data if they are not recorded. One example approach is to couple dynamical systems and perturbation techniques; a recent motor learning study demonstrated that disrupting activity in one brain region can provide insight into the computations performed by another recurrently-connected brain region, despite not directly observing the activity of that second region^29^. Future dynamical models will need to not only recapitulate multi-area observations, but also respond to causal manipulations in a consistent manner. Third, it will be important to disentangle the role of feedforward and feedback connections. Finally, current approaches assume linear correlations in neural state between areas, an assumption that may need to be relaxed.

A related approach likely to be of importance for modeling multi-area distributed computations is to model nonlinear dynamical systems via neural networks. Recurrent neural networks (RNNs) have been successfully used to model the dynamics of local, single-area computations in cognitive^30,31^, timing^32^, navigation^33^, and motor^6,34^ tasks. Following optimization (e.g., with backpropagation-through-time), RNNs often have synthetic activity that resembles electrophysiological activity, although if not, regularizations or other training techniques can typically be applied to induce strong resemblance to neurophysiological recordings^6,35^. This enables the RNN to act as an in silico model of the cortical area, where the RNN can be probed to propose dynamical hypotheses for the neural computations appearing in an area^30^. It is worth noting there exists a realism gap between RNNs and neural circuits. RNNs typically model network rates instead of spikes, use deterministic weights in place of dynamic synaptic connectivity, and are occasionally unconstrained in architecture. An important area of future research is to bridge the realism gap by determining what features of neural circuit computation can and cannot be abstracted in RNNs, which involves comparisons to data and testing of RNN proposed hypotheses. Another concern may be that RNNs will converge to different solutions based on experimenter-chosen hyperparameters, like the size of the networks, the machine learning hyperparameters of training, or other features. Intriguingly, a recent study suggests that key dynamical features, including fixed point structure, are robust to hyperparameter variation^36^.

One modeling opportunity to account for brain-wide computation is to expand RNN models to be multi-area, modeling within-area dynamics and inter-area communication (Fig. 2b). RNNs can be straightforwardly extended to incorporate multiple areas^31^ at a resolution that enables incorporation of anatomical constraints, including E–I cell types, proportion of connectivity between areas, and Dale’s law^37^. In these models, distinct RNNs can be treated as brain areas, with interactions defined by connections that implement CSs. While recent studies have similarly found that optimization can lead to RNN areas resembling brain areas^26,37^, training multi-area RNNs to resemble brain areas may be challenging and require additional training considerations, such as regularizing neural population trajectories of each RNN area to resemble cortical areas. Multi-area RNNs may propose hypotheses for how within-area dynamics perform computation and how inter-area connections selectively propagate upstream activity as inputs to downstream areas^37^. Extension of RNN modeling tools to multiple areas may therefore be an excellent candidate for generating new hypotheses for how behavior is shaped through distributed computation across multiple cortical areas.

Neural population dynamics offer a principled approach to the study of how neural circuits distributed across many brain areas orchestrate motor and cognitive function. We believe there are rich experimental and modeling opportunities to further our understanding of how multiple areas coordinate their dynamics to produce behavior.
